# An evaluation of a common elements treatment approach for youth in Somali refugee camps

**DOI:** 10.1017/gmh.2018.7

**Published:** 2018-04-25

**Authors:** L. K. Murray, B. J. Hall, S. Dorsey, A. M. Ugueto, E. S. Puffer, A. Sim, A. Ismael, J. Bass, C. Akiba, L. Lucid, J. Harrison, A. Erikson, P. A. Bolton

**Affiliations:** 1 Department of Mental Health and International Health, Johns Hopkins Bloomberg School of Public Health, Baltimore, Maryland, USA; 2 Global Community Mental Health Research Group, Department of Psychology, Faculty of Social Science, University of Macau, Macau (SAR), People's Republic of China; 3 Department of Health, Behavior, and Society, Johns Hopkins Bloomberg School of Public Health, Baltimore, Maryland, USA; 4 Department of Psychology, University of Washington, Seattle, Washington, USA; 5 Department of Psychiatry and Behavioral Sciences, McGovern Medical School at The University of Texas Health Science Center at Houston (UTHealth), Houston, Texas, USA; 6 Department of Psychology and Neuroscience and Duke Global Health Institute, Duke University, Durham, North Carolina, USA; 7 Department of Social Policy and Intervention, Centre for Evidence-Based Intervention, University of Oxford, Oxford, UK; 8 International Rescue Committee, IRC, Addis Ababa, Ethiopia; 9 Senior Technical Advisor, International Rescue Committee, New York, New York, USA

**Keywords:** transdiagnostic, implementation science, youth, refugee, cognitive behavioral therapy, interventions

## Abstract

**Background.:**

This paper reports on: (1) an evaluation of a common elements treatment approach (CETA) developed for comorbid presentations of depression, anxiety, traumatic stress, and/or externalizing symptoms among children in three Somali refugee camps on the Ethiopian/Somali border, and (2) an evaluation of implementation factors from the perspective of staff, lay providers, and families who engaged in the intervention.

**Methods.:**

This project was conducted in three refugee camps and utilized locally validated mental health instruments for internalizing, externalizing, and posttraumatic stress (PTS) symptoms. Participants were recruited from either a validity study or from referrals from social workers within International Rescue Committee Programs. Lay providers delivered CETA to youth (CETA-Youth) and families, and symptoms were re-assessed post-treatment. Providers and families responded to a semi-structured interview to assess implementation factors.

**Results.:**

Children who participated in the CETA-Youth open trial reported significant decreases in symptoms of internalizing (*d*  =  1.37), externalizing (*d*  =  0.85), and posttraumatic stress (*d*  =  1.71), and improvements in well-being (*d*  =  0.75). Caregivers also reported significant decreases in child symptoms. Qualitative results were positive toward the acceptability and appropriateness of treatment, and its feasibility.

**Conclusions.:**

This project is the first to examine a common elements approach (CETA: defined as flexible delivery of elements, order, and dosing) with children and caregivers in a low-resource setting with delivery by lay providers. CETA-Youth may offer an effective treatment that is easier to implement and scale-up versus multiple focal interventions. A fullscale randomized clinical trial is warranted.

## Introduction

Children, defined as any boy or girl under 18 years, make up almost half of the world's refugee population (United Nations High Commissioner for Refugees, 2016) and are exposed to challenges, traumas and stressors at the individual, family, and community levels that increase their risk for mental health problems (Barenbaum *et al.*, 2004; Betancourt & Khan, 2008; Reed *et al.*, 2012). These may include forced migration, forced labor, witnessing of murder and mass killings, lack of food and shelter, rape, torture, loss and separation from family, recruitment and use by armed forces, physical abuse, and family and sexual violence (Lustig *et al.*, 2004; Office of the Special Representative of the Secretary-General for Children & Armed Conflict *et al.*, 2009). Overlaid on potentially traumatic events are the daily, chronic stressors caused by displacement and associated with living in the camp environment – lack of basic needs, crowded and unsafe living conditions, and interpersonal conflict, among others (Layne *et al.*, 2010; Miller & Rasmussen, 2010).

Children affected by these types of traumas and stressors present with a wide range of mental health symptoms, including those associated with posttraumatic stress (PTS), depression, anxiety, conduct problems, risk behaviors (e.g., alcohol or drug use), and distress associated with grief reactions (Barenbaum *et al.*, 2004; Lustig *et al.*, 2004; Sagi-Schwartz, 2008; Attanayake *et al.*, 2009; Okello *et al.*, 2013[for review], Morgos *et al.*, 2008; Fernando *et al.*, 2010; Miller & Rasmussen, 2010; Reed *et al.*, 2012; Meyer *et al.*, 2013; Newnham *et al.*, 2015). Prevalence is difficult to study in these contexts; however, most studies find higher rates of mental health problems among displaced children compared with non-displaced populations (Goldstein *et al.*, 1997; Paardekooper *et al.*, 1999; Tousignant *et al.*, 1999; Morgos *et al.*, 2008; Bronstein & Montgomery, 2011; Reed *et al.*, 2012).

There remains limited evidence on the effectiveness of interventions to treat mental health problems of children in refugee settings in low- and middle-income countries (LMICs). Some studies suggest that cognitive–behavioral-based treatments can be effective for reducing mental health symptoms in refugee children (Bolton *et al.*, 2007; Layne *et al.*, 2010). Relatedly, some eclectic psychosocial programs have been tested with conflict-affected populations, and show an impact on outcomes such as self-esteem or hope, but not mental health (Tol *et al.*, 2014). Studies have generally utilized focal disorder treatments – or treatments focused on one disorder or cluster of symptoms. For example, Layne and colleagues used a treatment developed for posttraumatic stress disorder (PTSD) and grief (Layne *et al.*, unpublished treatment manual) and Bolton and team (2007) used a treatment developed for depression (Interpersonal Therapy for depression; IPT). In contrast to this focal disorder treatment model where everyone receives the same elements in the same order, in common elements approaches providers learn elements that can be combined in different ways (elements used, order, dose) to treat a range of common mental health symptoms, and how to handle comorbidity (e.g., depression, trauma, anxiety co-occurrence) (Chorpita *et al.*, 2005; Weisz *et al.*, 2011; Weisz *et al.*, 2012; Murray *et al.*, 2014*a*). This approach is potentially more efficient and sustainable in both high-income countries (Mansell *et al.*, 2008; Weisz *et al.*, 2011; Weisz *et al.*, 2012) and in LMICs (Bolton *et al.*, 2014; Murray *et al.*, 2014*a*; Ventevogel, 2015; Weisz *et al.*, 2015) because it does not require providers to learn multiple interventions. Additionally, in settings with high rates of disorder comorbidity, such as in refugee populations, a model that can address multiple disorders may also be more appropriate (Murray & Jordans, 2016).

## Current project

This paper reports on: (1) an evaluation of a common elements treatment approach (CETA) (Bolton *et al.*, 2007; Bolton *et al.*, 2014; Murray *et al.*, 2014*a*; Weisz *et al.*, 2015) developed for comorbid presentations of depression, anxiety, post traumatic stress, and/or externalizing symptoms among children in three Somali refugee camps on the Ethiopian/Somali border; and (2) an evaluation of implementation factors including acceptability, applicability/fit, feasibility and treatment facilitators, and barriers from the perspective of staff of the implementing partner organization, lay providers, and families who engaged in the intervention.

## Method

### Setting

This project was conducted in three refugee camps in the Somali region of Ethiopia: Kebri Beyah, Sheder, and Aw Barre, all near Jijiga town in the Ogaden region of eastern Ethiopia. Studies specifically on Somali refugee children are few, though a 2003 study estimated the prevalence of PTSD to be approximately 48% among Somali children and adults living in a refugee settlement in Uganda (Onyut *et al.*, 2009). As of September 2013, approximately the midpoint of the project, the combined total population of these three camps was 41,705 people, approximately 60% of whom were children under the age of 18 (United Nations High Commissioner for Refugees, 2014).

This project was completed within the context of regular programming of the partner organization, the International Rescue Committee (IRC) and had approval from the Ethiopian ARRA (Administration for Refugee and Returnee Affairs).

### Preceding qualitative and instrument validation studies

It is critical to understand local idioms of symptoms and validate local instruments when working cross-culturally (Bass *et al.*, 2007; Betancourt *et al.*, 2009; Kohrt *et al.*, 2011). This was done using a research and program development model, the Design, Implementation, Monitoring, and Evaluation (DIME) that aims to: (1) identify and measure local mental health problems through qualitative methods; (2) guide the selection, adaptation, and testing of mental health instruments and interventions; and (3) monitor and evaluate provided services in collaboration with local providers and community organizations (Applied Mental Health Research (AMHR) Group). Our qualitative study within these camps showed children exhibit a wide range of internalizing and externalizing symptoms (Puffer *et al.*, 2011). Further, we found the community was concerned about children who have experienced trauma – sexual violence, female genital mutilation, and other forms of abuse, as well as exposure to war-related violence. One Somali term for experiences of traumatic stress is ‘didsan,’ literally translated as ‘shock’ and described by the Somali team as referring to ‘a person who fears everything because they remember bad experiences.’

Based on results from the initial qualitative study, we adapted and validated three instruments (described below) to measure externalizing and internalizing symptoms (Hall *et al.*, 2014). The instruments were translated into the Somali language by a local translator hired by IRC who conducted back translation (into English), and all individual items were discussed by a local team of interviewers to assess the meaning, conceptual clarity of the translations, and cultural appropriateness of each item for the local context. Following translation, the measures were adapted by including additional items assessing locally relevant symptoms of internalizing, externalizing, and traumatic stress initially derived from a qualitative study (Puffer *et al.*, in progress). Results from a brief validation study indicated adequate psychometric properties for all three measures (see summary below; Hall *et al.*, 2014).

### Measures

#### Internalizing and externalizing symptoms

The Achenbach Child Behavior Checklist (CBCL)/Youth Self Report (YSR) (Achenbach & Rescorla, 2001) measured caregiver and child self-reported internalizing and externalizing symptoms. Respondents reported their frequency of experiencing each symptom, with responses coded on a three-point Likert-type scale ranging from ‘0’ (not true) to ‘2’ (very often true). The CBCL/YSR has evidenced excellent internal reliability in previous studies in the USA (Crijnen *et al.*, 1997; Ivanova *et al.*, 2007; Berubé & Achenbach, 2010), LMIC (Murray *et al.*, 2015), and during the validation study for this project (Hall *et al.*, 2014). The reliability coefficients for caregivers and children were 0.95 and 0.88 for the Internalizing Scale, 0.89 and 0.93 for the Externalizing Scale, respectively.

#### PTS symptoms

Child Post Traumatic Stress Disorder Symptom Scale-Interview format (CPSS-I) (Foa *et al.*, 2001) assessed 17 PTS symptoms on a four-point Likert-type scale ranging from ‘0’ (never) to ‘3’ (all the time). The CPSS has evidenced excellent psychometric properties, with Cronbach's alpha (*α*) and test–retest values exceeding 0.80 (Foa *et al.*, 2001). Child and caregiver forms of the instrument assessed the child's symptoms. Although the CPSS-I does not have a caregiver version, the scale was adapted to assess caregiver report of children's symptoms. The internal reliability obtained in the validation study for both caregivers and children was 0.95, and 0.85, and combined test–retest and interrater reliability was reasonable for caregivers (*r*  =  0.72) and children (*r*_s_  =  0.45) (Hall *et al.*, 2014). Cronbach's *α* was 0.94 for caregivers and children in the current study.

#### Child well being

The Orphans and Vulnerable Children Wellbeing Tool (Senefeld *et al.*, 2009) measured aspects related to child well-being. Sample items included ‘I am as happy as other kids my age,’ ‘I feel secure in my neighborhood,’ and ‘My body is physically healthy.’ This instrument was developed for use with orphans and vulnerable children. The present project utilized a 31-item version of the instrument (reduced from 36 items), which was psychometrically evaluated in the validity study (as above), and shown to have a high reliability and combined test–retest and interrater reliability for children, but not for caregiver report. Therefore, the instrument was only used to measure children's self-reported well-being. The scale had excellent reliability in the present project (Cronbach's *α*  =  0.94).

We established cut scores for project screening purposes by following the results of the validity study (Hall *et al.*, 2014). The cut score of 13 was chosen for traumatic stress symptoms based on ROC (receiver operating characteristic curve) analysis demonstrating this as an acceptable score to balance specificity and sensitivity. Cut scores for internalizing and externalizing symptoms were not considered reliable based on ROC analysis so the project team utilized a score of 19 or higher for these symptom dimensions based on maximizing the sensitivity of symptoms. This cut-off required a response of ‘sometimes’ or greater on at least half the items.

#### Project measure procedures

The three measures, together with the demographic information, were compiled into a complete project instrument. For demographics, age, sex, and educational status were obtained for 37 caregiver and child dyads. For caregivers, marital status, spousal cohabitation, and the length of time living in Ethiopia also were obtained. Participants and their caregivers were administered the project instrument prior to beginning the intervention and 1 month following completion of the counseling program.

### Recruitment, consent, and baseline assessment procedures

This project was conducted from January 2013 to February 2014. Participants were recruited with two methods: from the validity study population (*N*  =  16), or from referrals [social workers within IRC who knew families in need (*N*  =  19), and general referral (*N*  =  2)]. Three IRC staff and 17 refugee camp-based workers were trained to administer the project instrument as well as deliver the intervention as part of IRC programming.

First, project staff reviewed the list of participants from the prior instrument validation study [described above (Hall *et al.*, 2014)] whose scores indicated elevated symptoms as per the inclusion criteria. A local IRC staff member who functioned as the project manager (AI) completed a cover sheet on all validity study participants that met inclusion criteria. This cover sheet contained identifying information including the child's name, age, address, and their study ID number from the validity study; the cover sheet did not include information on the symptom problems or any other study-related data. When referrals were made by social workers in the IRC's child protection program, the IRC project manager (AI) compared the referral list against the list of children from the validity study to ensure there would be no duplication in screening. A cover sheet was completed in the same process for all referred children – each with a unique ID identifier.

The cover sheets were given to IRC staff and camp-based workers who went into the community to identify the children, describe the project and complete informed consent procedures. If there was a discrepancy between the identification information on the cover sheet and information provided by the individual, the IRC workers did not proceed with informed consent and reported back to the project manager. If the subject's identity was successfully verified, the IRC workers obtained oral informed consent from the child's caregiver (required for inclusion; defined as the person primarily responsible for the child) and informed assent from the child using a standard script. Caregivers had to give permission prior to an assessor speaking with a child. IRC workers obtained informed consent and assent from caregivers and children (separately) for screening and, if eligible, proceeded to read a second consent form to participate in the treatment and evaluation (again, separately). All interviews were conducted in confidential locations (e.g., IRC offices, clients' homes) and at a time of the participant's choosing. All children in the project had to present with a caregiver to assure proper adult consent.

**Fig. 1.**
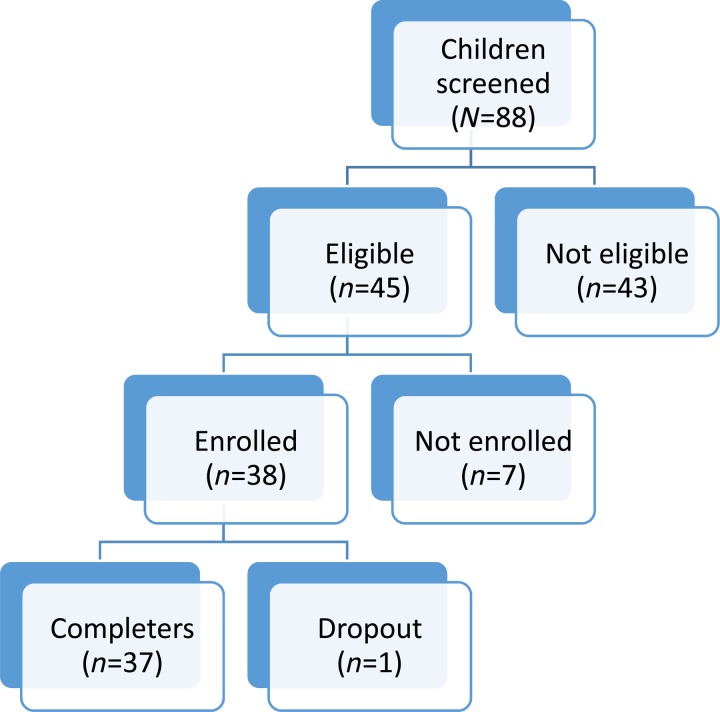
Flowchart of participants.

Participant characteristics are displayed in Table 1. A total of 88 children were screened for participation in the evaluation of CETA (see Fig. 1). Inclusion criteria for the children were being between 7 and 18 years of age, living within one of the three identified camps, and elevated symptoms in at least one of the domains of mental health problems identified in the prior qualitative study: (a) trauma-related symptoms as assessed by the adapted CPSS, cut score 13, (b) externalizing symptoms as assessed by the adapted CBCL/YSR externalizing scale, cut score 19, and/or (c) internalizing symptoms as assessed by the adapted CBCL/YSR internalizing scale, cut score 19. As the treatment model was a common elements approach that could be used for children with comorbidities, participants could meet inclusion by any single or multiple cut-off scores. Children were excluded from the project if the child or their legal caregiver was not mentally competent to give consent, or if the child was the head of household.

**Table 1.** Table of Elements in CETA-Youth

Element	Simplified Name (Used in training)	Description
Engagement	Encouraging Participation (done in same session as Introduction)	Focus on obstacles to engagement Link program to assisting with client's problems Include family when appropriate
Psychoeducation	Introduction (done in same session as Encouraging Participation)	Give program information (duration, content, expectations) Normalize/validate current symptoms/problems
Parenting Skills (Caregiver only)	Caregiver skills	Provide positive one-on-one time, praise, reward and special thanks, giving effective commands, consequences
Anxiety management strategies	Relaxation	Teach strategies to improve physiological stress Examples include: deep breathing, meditation, progressive muscle relaxation, and imagery. Others added based on local cultures.
Behavioral Activation	Getting Active	· Identify and engage in pleasurable, mood-boosting, or efficacy-increasing activities
Cognitive Coping/Restructuring	Learning about Thoughts Feelings and Behaviors Thinking in a Different Way Part I Thinking in a Different Way Part II	Understand what thoughts feelings and behaviors are. Understand connection between thoughts, feelings, and behaviors with benign situations. Learn to restructure thinking to be more accurate and/or helpful.
Imaginal Gradual Exposure	Talking about Difficult Memories Final chapter on what they learned and how they are different	Face feared and avoided memories in detail Gradual desensitization/exposure
In Vivo Exposure	Live Exposure	Face innocuous triggers/reminders in the client's environment Gradual desensitization/exposure
Problem Solving	Problem Solving	Defining a problem, brainstorming solutions, understanding pros and cons of the solutions, choosing a solution to try, and breaking that solution into smaller steps.
Suicide/Homicide/Danger Assessment and Planning	Safety	Assess client risk for suicide, homicide, and domestic violence Develop a focused plan with the client and client's family (when appropriate) Additional referral/reporting when needed

Post-assessments were administered by an IRC staff member that did not provide treatment to the child/family. These assessments were given to children and caregivers regardless of the number of CETA sessions completed.

### Safety protocol

Interviewers were trained to identify interview responses that might indicate a child was at immediate risk of harm, including suicide, homicide, serious violence to self or others, and physical, sexual or psychological abuse. If such risks were identified, a locally-developed safety protocol was implemented (Murray *et al.*, 2014*b*).

### Intervention

The Common Elements Treatment Approach for youth (CETA-Youth; Table 1) was developed by two authors (LKM, SD), based on other common elements or transdiagnostic treatment research from the USA (Chorpita & Weisz, 2009a, b; Weisz *et al.*, 2012; Chorpita *et al.*, 2017), and designed specifically for training and delivery by non-professional, lay providers, in low-resource settings (Murray *et al.*, 2014*a*). CETA is an approach that teaches cognitive-behavioral therapy elements common to evidence-based treatments (EBTs) (Chorpita *et al.*, 2005) for trauma, anxiety, depression, and behavioral problems, and also how to combine these elements to treat different presenting problems and comorbidity. Our team conducted randomized clinical trials of CETA with adults in two LMIC settings. Both studies incorporated a task sharing approach where most providers had limited education and little to no formal mental health training and were taught by the apprenticeship model (Murray *et al.*, 2011). The studies showed medium to large effect sizes including: (a) on the Thailand/Myanmar border with Myanmar refugees (*N*  =  347; ES: 1.19 PTS, 1.16 depression, 0.79 anxiety) (Bolton *et al.*, 2014) and (b) in Southern Iraq with survivors of conflict, torture, trauma, and ongoing stressors (*N*  =  149; ES: 2.40 PTS, 1.82 depression, 1.60 anxiety) (Weisz *et al.*, 2015). These findings provide some evidence of effectiveness for CETA with adults, as well as the ability of lay providers to learn this type of flexible approach.

Suggested implementation for CETA-Youth was weekly 60–90- minutes sessions with the child and caregiver (if available), and involved delivery of varying elements and ‘dose’ of elements (i.e. the number of sessions or time spent on element) depending on client symptom and need. The number of sessions provided could vary, with a suggested 6–12 sessions depending on need. If the caregiver was involved, they were taught the same skills as the child so they could support the child. When the child had behavior problems, the caregiver was also taught parenting skills.

Counselors were taught how to identify a primary problem by considering three things: (1) assessment forms, (2) what the child/caregiver does and says, and (3) consultation with their supervisor. For assessment, the counselors were taught to complete an Elements Decision Making Table, which grouped items from the assessment focused on one problem area (e.g., sadness or trauma). For example, a child may score 9/12 on items related to trauma (e.g. avoidance, nightmares), and only 3/12 for items related to depression (e.g. crying, not doing activities that bring pleasure). During the first session, counselors would gather more information about what is bothering the child and caregiver the most. In supervision after the first session, a flow would be determined collaboratively with the counselor and supervisor, and then would be passed on to the trainer for approval. The counselors were taught example or ‘default’ flows for primary problems. These ‘default’ flows were based on current EBTs for a particular problem area. For example, if a primary problem is trauma-related, the default flow of elements is: Introduction, Learning, Thinking in a different way - Part 1 (i.e., cognitive coping), Talking about difficult memories (imaginal exposure), Thinking in a different way part 2 (cognitive processing), and Finishing. Lay counselors were taught to provide a second session whenever they had not completed all the steps for an element, when they believed a client did not learn the element, when a client did not complete his/her homework and needed practice, or when counselors felt that symptoms had not improved sufficiently (by client or caregiver report in session or on the weekly monitoring form). ‘Default flows’ were created so that decision making was relatively easy for lay providers with no formal training. In this way, they could learn to recognize a primary problem and suggest a default flow. During the in-person training, counselors practiced decision making with a wide range of vignettes that included questions about a client's progress in a session or homework completion. Counselors worked in small groups to think through answers for the next session (e.g., move to the next element or provide another dose of the current element) while trainers rotated through the small groups. In this way, counselors and supervisors developed CETA decision-making skills. These skills continued to be developed during practice groups and group-based supervision (supervision of their own clients and clients seen by their peers).

Nineteen lay counselors and three IRC local supervisors were trained in CETA. All local counselors were fluent in Somali and chosen based on their verbal communication skills and expressing a desire to gain counseling skills and work with children. Experience in either counseling or working with children was preferred but not required. Backgrounds varied with most having some secondary education. Three of the counselors were trained by IRC previously in providing case management. Supervisors were staff from the IRC's gender-based violence and child protection programs; all had undergraduate college degrees in social work or nursing but no formal mental health training.

Counselors and supervisors were trained in CETA by US-based clinical psychologists (CETA-Youth) utilizing the apprenticeship model of training and supervision (Murray *et al.*, 2011) which consisted of a 10-day in-person training, followed by 6 months of weekly small group meetings in which lay counselors either practiced the treatment elements with a local supervisor (before they were providing CETA to clients) or received supervision on each case (once they began providing CETA to clients). Weekly Skype meetings were also held between each local supervisor and a CETA trainer (LKM and AMU) for 1–2 h to either review group role-plays or discuss CETA cases. When internet connection would not allow a skype call, the trainers either emailed the supervisors or called their mobile phones to discuss.

### Intervention fidelity

Prior to treatment initiation, counselors and local supervisors documented CETA treatment elements they thought should be delivered for each client and in what order, and the local supervisor reviewed this with a CETA trainer (LKM, SD, AMU). Once an initial order was agreed upon, counselors proceeded and each week documented how they provided each element according to specific steps detailed in the CETA-Youth manual. Supervisors elicited details from lay counselors, reviewed materials from in-session activities, and recorded the techniques used and homework assigned during weekly supervision meetings. If an element (e.g., Thinking in a Different Way), or element ‘step’ was missed (e.g., assigning homework), the supervisor requested completion in the next session. In each supervision session, the local supervisor and counselor discussed and/or role-played the element and planned for the next session. A CETA trainer (LKM and AMU) recorded detailed notes from the supervisors’ weekly verbal reports, checking that all CETA elements were delivered with proper technique, or if not, asked for those elements to be re-done in the following session. This was done through asking for an objective reporting of what happened in session, answering questions, and/or role-playing with supervisors (to understand what happened and/or demonstrate how a skill should be implemented).

### Qualitative evaluation of CETA

To assess implementation aspects of the project (e.g., feasibility, acceptability, appropriateness for problems, barriers, and facilitators), the IRC and academic research team collaboratively developed brief semi-structured interview guides for children and caregivers, counselors, and supervisors. For children and caregivers, counselors who did not provide treatment for the family completed each interviews in pairs. Four broad, open-ended questions were asked about participants’ perceptions of how the counseling led to change in the child and caregiver, including a probe about potential changes in relationships: participants’ description of their experiences in counseling; and participants’ likes and dislikes about counseling. Interviewers were trained to use very general probes to ensure participants had the opportunity to list as many responses as they wished. These interviews were conducted in one to three 1-hour-long sessions, with the academic research team and IRC team reviewing transcripts in between interviews to identify additional interview probes as needed.

For the counselor and supervisor interviews, the IRC hired two local interviewers not otherwise involved in the project. These two staff received a 1-day interactive training on qualitative interviewing techniques conducted by IRC staff. These interviews were conducted in a single, 1-hour session. Interviewers asked counselors broad, open-ended questions covering the following topics: their experiences providing counseling, including probes related to positive and challenging experiences; their description of becoming a counselor, including positive and challenging aspects of this new role; their description and opinions related to the training and supervision process; and their reflections on the fit of the counseling with the context of the refugee camps. All qualitative interviews were audio-recorded, transcribed in Somali, and then translated into English to enable both local and international IRC staff and research team partners to participate in review and analysis.

## Analysis plan

### Differences in baseline sample characteristics

We compared treatment participants and eligible treatment non-participants to determine whether demographic characteristics varied by group. Paired sample *t* tests for normally distributed continuous outcomes and Wilcoxon Rank-sum tests for non-normally distributed continuous baseline measurements of treatment outcomes were used to test mean baseline differences between treatment completers (*n*  =  37) and treatment non-participants (*n*  =  8) to assess for potential non-participation bias.

Baseline demographic characteristics (i.e. child's age, sex, school status, education level, caregiver's age, sex, and whether they were cohabitating with a spouse) were compared between eligible treatment participants and treatment non-participants using independent samples *t* tests, chi-square tests, and Fisher's exact test for variables with fewer than five observations in any cell.

### Quantitative analysis of main outcomes

To evaluate whether treatment completers experienced statistically significant changes from pre- to post-treatment, we conducted paired sample *t* tests for normally distributed outcomes and Wilcoxon Rank-sum tests for non-normally distributed outcomes. Outcomes included changes in internalizing, externalizing, and PTS symptoms as reported by caregivers and children, and child well-being as reported by children. Effect sizes were calculated (Cohen's *d* for paired sample *t* tests and *r* for Wilcoxon Rank-sum tests) and interpreted such that for Cohen's *d*, 0.20  =  small, 0.50  = medium, and 0.80  = large effect; and for *r*, 0.10  = small, 0.30  = medium, and 0.50  = large effect (Cohen, 1992).

### Qualitative analysis of implementation and trial results

To analyze the qualitative data gathered from the four types of participants (caregivers, children, providers, and supervisors), transcripts were translated into English from Somali by local bilingual staff. Coders (CA, LL, JH) read the transcripts to establish a general understanding of the material. Passages in the text were coded in Microsoft Excel using an open coding procedure by the three independent coders to identify the codes and count frequencies. The codes were regularly reviewed throughout the coding process by the three coders and any disagreements were discussed with a fourth member of the research team (CA, LL, JH, LM). Thematic coding continued iteratively until all of the transcripts had been coded and discussed resulting in the identification of major and minor themes.

## Results

### Treatment participants

Of the 45 children eligible for treatment, eight (17%) were not enrolled as they declined participation due to physical health problems (*n*  =  4) or moving away from the camp (*n*  =  4). Thirty-eight children (83%) enrolled in the project Table 2. All but one child completed both the pre and post-treatment assessment (this child moved from the camp).

**Table 2.** Sample characteristics for Children and Caregivers (N = 37).

	Child	Caregiver
Age (M (SD))	11.21 (3.17)	41.24 (11.83)
Sex (% female)	44.44	82.86
Education*		
None	21.88	57.14
Some or graduated primary	62.49	22.85
Middle school	3.12	8.57
Some high school	12.5	2.86
High school graduate or higher	−	8.57
Number of years living in the camps (M (SD))	−	8.59 (8.19)
Marital Status (% married or in relationship)	−	83.33
Living with spouse (% cohabitating)	−	58.82

*Current grade for children; Highest grade for caregivers

Children who were eligible but did not participate were significantly different on child age (*M*_non-participants_  =  13.75, s.d.  = 2.31 *v*. *M*_participants_  = 11.21, s.d. 3.18, *p*  = 0.043) and caregiver spousal status (Fisher's exact test, *p*  =  0.045), with the caregivers of non-participants reporting not having spouses at home with greater frequency than caregivers of treatment participants. No additional statistically significant differences for child and caregiver characteristics were noted across the two groups.

All children were included in the analysis, except for one that did not complete the post-treatment assessment. The majority screened positive for comorbid PTS and internalizing problems (51.35%). Among the children who participated, the remaining children screened positive for comorbid PTS, internalizing and externalizing (18.92%), PTS only (13.51%), externalizing only (8.11%), internalizing only (5.41%), and comorbid PTS and externalizing (2.70%).

### Outcomes

#### Internalizing symptoms

Children reported reduced average internalizing symptom scores from baseline (*M*  =  25.73, s.d.  =  1.97) to post-treatment (*M*  =  7.76, s.d.  =  1.66); *t*_(36)_  =  8.35, *p* < 0.001, with a large effect size (Cohen's *d*  =  1.37). Caregivers also reported a significant decrease in internalizing symptoms from baseline (*Mdn*  =  12) to post-treatment (*Mdn*  =  3); *Z*  =  4.965, *p* < 0.001, with a large effect size (*r* = 0.85) Table 3.

**Table 3.** Pre-post mean scores for treatment completers (N = 37).

Child scales	Baseline	Follow-up	t or z statistic†	*p*	Effect size *(d* or *r)*
Achenbach: Internalizing with qualitative items	25.73 (12.01)	7.77 (10.14)	8.35	<.0001	1.37
Achenbach: Externalizing with qualitative items	13.63 (7.33)	3.58 (5.31)	4.96†	<.0001	0.82
CPSS-I with qualitative items	20.84 (8.69)	5.57 (5.89)	10.39	<.0001	1.71
Child well-being	47.48 (15.98)	62.72 (15.34)	4.58	=.0001	0.75
**Caregiver scales**					
Achenbach: Internalizing with qualitative items	20.65 (8.26)	8.00 (9.24)	4.97†	<.0001	0.82
Achenbach: Externalizing with qualitative items	16.41 (10.99)	4.86 (7.02)	4.63†	<.0001	0.76
CPSS-I with qualitative items	21.19(8.27)	7.45 (9.55)	5.17†	<.0001	0.85

*Note*. CPSS-I = Child PTSD Symptom Scale-Interview. †Based on Wilcoxon Rank-sum tests for non-normally distributed variables. Effect size interpretation: Cohen's *d:* .02 = small; .50 = medium; .80+ = large effect. *r:* .10 = small; .30 = medium; .50 = large effect.

#### Externalizing symptoms

Children reported significant decrease for average externalizing symptom scores from baseline (*Mdn*  =  7.5) to post-treatment (*Mdn*  =  1); *Z*  = 4.958, *p* < 0.001, with a large effect size (*r* = 0.85). Significant decreases were also observed for caregiver-reported externalizing symptoms from baseline (*Mdn*  =  7) to post-treatment (*Mdn*  =  2); *Z*  = 4.625, *p* < 0.001, with a large effect size (*r*  = 0.82).

#### Trauma symptoms

Children reported a reduction in average PTS symptom scores from baseline (*M*  =  20.83, s.d.  =  8.69) to post-treatment (*M*  =  5.56, s.d.  =  5.89); *t*_(36)_  =  10.38, *p* < 0.001, with a large effect size (Cohen's *d*  =  1.71). Caregivers similarly reported a significant decrease in children's PTS symptoms from baseline (*Mdn*  =  11) to post-treatment (*Mdn*  =  4); *Z*  =  5.168, *p* < 0.001, with a medium to large effect size (*r*  =  0.76).

#### Secondary outcome

*Child well-being*. Significant increases in average child-reported child-wellbeing scores were observed from baseline (*M*  =  47.48, s.d.  =  15.98) to post-treatment (*M*  =  62.72, s.d.  =  15.34); *t*_(4)_ = −4.58, *p* < 0.001, with a medium to large effect size (Cohen's *d*  =  0.75).

### Clinical implementation results

Participants that were analyzed (*n*  =  37) (those that had both pre and post assessments) averaged 8.81 sessions (range: 4–13). Thirty-one participants were classified as getting an ‘adequate dose’ of CETA, defined as having received at least one session of the following elements: Introduction/Encouraging Participation, Learning, Thinking in a Different Way Part 1, Talking about difficult memories, and Thinking in a Different Way Part 2. For these 31 participants, the average number of sessions was 9.42 with a range of 6–14. The reasons for the six cases not completing an ‘adequate dose’ of CETA were unknown. Five of the six dropped after the talking about difficult memories (TDM) element with low symptom scores and may have been feeling better. One case stopped coming after Talking about Difficult memories. CETA is designed with flexibility in session number, and completion is based on symptom reduction and receipt of the recommended elements.

Caregivers were engaged in most cases, attending an average of 9.42 sessions (range 6–13). The amount of time spent on each element across one or more sessions for children and caregivers are presented in Table 4. On average, the 17 counselors (two left the project) provided CETA to 2.29 participants (range: 1–4). Two high-risk cases were identified: both were successfully managed with the safety protocol.

**Table 4.** Average Amount of Time Counselors Spent Delivering Elements to Child & Caregiver

Element Name	Minutes Spent Delivering Element to Child (mean)	Minutes Spent Delivering Element to Caregiver (mean)
*Encouraging Participation & Introduction*	42.67	42.06
*Safety*	12.86	13.33
*Learning about Thoughts, Feelings, and Behaviors*	52.51	51.61
*Thinking in a Different Way – Part I*	69.15	53.58
*Talking About Difficult Memories*	124.88	83.08
*Thinking in a Different Way – Part II*	74.99	47.23
*Talking About Difficult Memories – Final Chapter*	45.75	40.68
*Getting Active*	48.31	38.18
*Relaxation*	57.50	44.33
*Praise*	30.00	42.50
*Problem Solving*	82.50	32.50
*Finishing*	35.79	39.62

### Qualitative results

Based on qualitative interviews with children who received CETA and their caregiver (*N*  = 35 of 37; two children moved away), CETA was described as acceptable and beneficial, with only a few reported child/caregiver concerns. Children reported liking the counseling offered in CETA, citing benefits that included general overall changes and improvements in behaviors and mood (*n*  =  30/35). Children also reported improvements in more specific areas related to thoughts, feelings, behaviors, as well as some trauma-specific improvements [less unhelpful thinking (*n*  =  14); fear/anxiety (*n*  =  16); anger/aggression (*n*  =  13); mood (*n*  =  15)]. Children reported good relationships with their friends, family, and community after counseling (*n*  =  18/35) and noted overall positive changes (*n*  =  10/35) in their relationships. When asked about their own experiences in counseling, child participants most frequently discussed the skills they learned, specifically how to change negative thoughts, how to calm themselves, being obedient, and integrating with the community. When asked about any parts of CETA-Youth that they did not like, children did not endorse any specific dislikes.

Caregivers reported overall changes and improvements in their children (*n*  =  26/35). Caregivers cited specific improvements related to thoughts, feelings, and behaviors [fear/anxiety] (*n*  =  19/35); mental/thinking (*n*  =  15/35); anger/aggression (*n*  =  19/35); and mood (*n*  =  10). Caregivers also reported that children had improved relationships with community members (*n*  =  21/35), their friends (*n*  =  20/35), and their families (*n*  =  18/35). Caregivers reported that children behaved more obediently (*n*  =  16/35) and respectfully (*n*  =  9/35) toward their family members, and offered help to family and neighbors (*n*  =  8/35). When asked about the counseling experience, caregivers discussed skills such as learning how to help children change maladaptive thoughts and how to help them feel calm. When asked about things they disliked about CETA-Youth, some caregivers said they did not like the personal nature of some of the questions in the assessments, such as ‘does your child use alcohol.’ They also reported wishing that they were provided with other services outside the scope of CETA (e.g. requested materials goods like blankets and sleeping maps, assistance with relocation).

Three IRC local supervisors and 13 of the 17 counselors also participated in qualitative interviews about their experience delivering CETA-Youth. The providers themselves (*n*  =  16) spoke about several facilitators, barriers, and solutions to implementation factors. The vast majority of providers reported the post-training practice groups and supervision helped to further their understanding of the intervention material (*n*  =  14/16), particularly during group role-plays, which are key to the apprenticeship model (*n*  =  5/16). Other providers spoke directly about the content of the training itself stating that it was most useful to learn about the intervention in terms of its components (*n*  =  8/16), particularly the connection among one's thoughts, feelings, and behaviors (*n*  =  8/16). Some counselors also spoke about the importance of being trained on how to match a client's symptoms with specific intervention components (*n*  =  4/16). Providers reported being motivated by seeing positive changes in their clients once they began counseling (*n*  =  12/16) and feeling welcomed into the lives of their clients’ families (*n*  =  6/16). Finally, the providers discussed how having quality materials (e.g., manual and handouts) that outlined specific steps for the providers to take during each session allowed them to more easily deliver the intervention (*n*  =  6/16).

In terms of barriers and potential solutions the providers most commonly discussed the difficulties of participant engagement (*n*  =  11/16), particularly in terms of the caregivers (*n*  =  6/16) who they felt contributed to the client no-shows (*n*  =  7/16). Possibly linked with the low level of engagement, providers also mentioned how common it was for participants to expect benefits that fell outside the scope of counseling (e.g., blankets, sleeping mats, help with resettlement) (*n*  =  10/16). This phenomenon is perhaps best characterized by one provider who said that “Parents tell us many needs, they tell you a Somali proverb saying, ‘*a place in which take (physical things) is needed then talk is not helpful*’ that shows us that they need some things to be given to them in the hand during counseling.’ The providers suggested the provision of such materials in order to aid their counseling efforts (*n*  =  8/16). Some counselors also discussed how clients’ religious and cultural views may have impacted their level of engagement. Some providers noted that the general belief in the camps was that mental health issues came from a higher religious power (*n*  =  5/16) and that the most common practice to treat mental illness is to have the afflicted parties read religious scripture (*n*  =  8/16) making counseling a somewhat foreign form of treatment (*n*  =  3/16). Few counselors posed solutions to these issues, however one that was mentioned was to more clearly orient participants to the benefits of counseling services (*n* = 2/16).

## Discussion

The present project evaluated a common elements psychotherapeutic approach for conflict-affected Somali children and their caregivers residing in a refugee camp. Children who participated in the CETA-Youth project had decreases in child and caregiver-reported symptoms of internalizing, externalizing, and PTS, and improvements in well-being. Effect sizes for most outcomes were large, with the largest effect for PTS (nearly double the other effects).

This evaluation is the first to examine a common elements approach (defined as flexible delivery of elements, order, and dosing) with children and caregivers in a low-resource setting with delivery by lay providers. Two prior RCTs testing CETA with adults in LMIC (Bolton *et al.*, 2014; Weiss *et al.*, 2015) demonstrated effectiveness. However, CETA-Youth was adapted for a child/adolescent focus and incorporated additional elements to address externalizing symptoms and to incorporate parental involvement in the child's treatment. Changing the population focus to children, adding an element (i.e. parenting skills), and adding caregiver steps for each element (so caregivers could support their children) had potential to compromise lay counselors’ ability to learn the intervention and deliver it with fidelity. However, our results suggest that CETA-Youth can be delivered by lay providers when they receive active training, practice opportunities, and supervision (Murray *et al.*, 2011). Counselors, with supervision from local supervisors, were able to choose elements, their order, and dosing to include caregivers and address a range of comorbid symptomatology. This is important because comorbidity is the norm in most populations (Weisz *et al.*, 2015).

This was the first evaluation of CETA where the inclusion criteria were allowed to vary – meaning children could enroll if they had any of the symptoms but did not need any one in particular as most trials require (Bolton *et al.*, 2014; Weiss *et al.*, 2015). Children entered into the CETA program with a wide range of problems and severities, including internalizing, externalizing and comorbid problems. Over 70% had more than one presenting problem. Of these comorbid youth, 27% had three primary presenting problems. Our findings support research suggesting that comorbidity is the ‘norm and not the exception’ (Weisz *et al.*, 2015). Currently, most EBTs target one primary problem, with limited guidance when comorbidity is encountered. Increasingly, in high- and low-income countries transdiagnostic or common element approaches are being promoted as a potentially effective way to reach, scale-up, and sustain mental health services as they equip providers with skills to treat multiple presenting problems and handle comorbidity (Ventevogel, 2014; Murray & Jordans, 2016). This evaluation allowed us the unique opportunity to include a lower resource context in the relatively earlier stages of developing the evidence base for this relatively new approach in high-resource settings.

CETA-Youth was delivered in relatively few sessions (13 or fewer), which is consistent with the adult CETA trials (Bolton *et al.*, 2014; Weiss *et al.*, 2015) and constitutes a lower dose than in many EBT. For example, Trauma-focused Cognitive Behavioral Therapy (Cohen *et al.*, 2006), for children is manualized for 12–16 sessions; although a recent study in the USA with mental health professionals demonstrated effectiveness in eight 90-min sessions (Deblinger *et al.*, 2011). Another child-focused EBT, ‘Coping Cat’, is manualized for 16 sessions (14 individual and two parent) (Kendall, 1994; Kendall *et al.*, 1997; Silk *et al.*, 2016). This may also reflect the fact that fewer elements or doses may have been used for less symptomatic youth. The relatively few number of sessions (and thus time) appeared to work well for this transient population where most were seeking resettlement.

This evaluation is also unique in that CETA was implemented by the IRC, an agency already providing a range of psychosocial services in the camps. Related to the Inter-Agency Standing Committee (IASC) pyramid (Inter-Agency Standing Committee, 2007), IRC was already providing two ‘lower’ portions of prevention education and general support services (e.g. child-friendly spaces; case management for cases of child maltreatment/neglect). However, our qualitative study (Puffer *et al.*, 2011) indicated that this population identified significant mental health issues and spoke of the lack of services available. CETA-Youth was successfully incorporated so that IRC programming (and the same providers) offered multiple layers of the IASC pyramid. The addition of a common elements flexible approach is one way of rounding out the delivery of the range of psychosocial services to populations that are likely to have mental health needs (low, moderate, or significant) due to chronic traumas and stressors that may be common to such populations. In this way, the elements can be used to address the range of needs (low to significant) (Murray & Jordans, 2016) and potentially reduce the need for multiple different manuals/interventions.

Qualitative results suggest that overall CETA was accepted by participants. This is similar to findings from other CBT-based interventions in LMIC (Murray *et al.*, 2014*c*). Many commented on how much the program helped them, which suggests that the strong effect sizes are linked to acceptability. Providers of CETA in this project specifically commented in a positive way about the apprenticeship model of training and ongoing supervision. Although this model may present some challenges for scale-up due to the level of resources required, these results suggest that lay providers may need and appreciate this level of support when delivering a mental health program. The barrier to engagement due to clients needing tangible resources is a common struggle when implementing talk therapies in LMIC. Indeed, this type of engagement challenge is present in many low-resource populations (McDaid *et al.*, 2008). That said, the counselors were still able to engage families in treatment with high completion.

There are important limitations to this evaluation. First, this was an evaluation of a project with no randomization or control group. Therefore, alternative explanations for reductions in children's symptoms cannot be ruled out. Second, the sample size was small. This project was designed to address identified mental health needs within a population with which IRC was working, and the goal was to pilot the CETA-Youth approach and examine feasibility. Including a control group and having a larger sample will be necessary to empirically evaluate the effectiveness of CETA-Youth. Third, there was no follow-up to determine if treatment effects were sustained over time. Also, records of what elements were done in each session were based on a verbal report from the lay counselor to the local supervisor, and then onto the trainer. Finally, the qualitative interviews yielded relatively brief responses from youth and caregivers, rather than more in-depth narrative responses that could have provided a more comprehensive understanding of participants’ experiences and potential mechanisms of change.

## Conclusions and future directions

Conflict-affected children and adolescents living in refugee camps who received CETA-Youth had significant decreases in diverse mental health symptoms (PTS, internalizing, externalizing) and increases in well-being. Both child and caregiver participants reported high levels of acceptability and satisfaction with the intervention. Given the high comorbidity of our sample and high comorbidity among conflict-affected youth worldwide, findings from this evaluation are promising and suggest a need for more research rigorously testing the CETA model. Also promising, CETA-Youth was tested used a task-sharing approach in which lay counselors from the community delivered the intervention. This approach is in line with WHO recommendations for using task-sharing to deliver psychosocial treatments. To our knowledge, it is the first to test a common elements treatment with flexible use of elements and dose for children in LMIC. Given the high mental health treatment gap worldwide, particularly for children, and the ‘norm’ of comorbid presentations, CETA-Youth may offer an effective treatment that is easier to implement and scale-up versus multiple focal interventions, specifically for populations with high comorbidity.
